# Acute and Chronic Dosing of a High-Affinity Rat/Mouse Chimeric Transferrin Receptor Antibody in Mice

**DOI:** 10.3390/pharmaceutics12090852

**Published:** 2020-09-08

**Authors:** Demi M. Castellanos, Jiahong Sun, Joshua Yang, Weijun Ou, Alexander C. Zambon, William M. Pardridge, Rachita K. Sumbria

**Affiliations:** 1 Henry E. Riggs School of Applied Life Sciences, Keck Graduate Institute, Claremont, CA 91711, USA; dcastellano18@students.kgi.edu (D.M.C.); jyang16@students.kgi.edu (J.Y.); 2 Department of Biopharmaceutical Sciences, School of Pharmacy and Health Sciences, Keck Graduate Institute, Claremont, CA 91711, USA; Jiahong_Sun@kgi.edu (J.S.); Weijun_Ou@kgi.edu (W.O.); Alexander_Zambon@kgi.edu (A.C.Z.); 3 Department of Medicine, University of California, Los Angeles, CA 90095, USA; wpardrid@ucla.edu; 4 Department of Neurology, University of California, Irvine, CA 92868, USA

**Keywords:** transferrin receptor, transferrin receptor monoclonal antibody, iron, blood-brain barrier, pharmacokinetics, reticulocytes

## Abstract

Non-invasive brain delivery of neurotherapeutics is challenging due to the blood-brain barrier. The revived interest in transferrin receptor antibodies (TfRMAbs) as brain drug-delivery vectors has revealed the effect of dosing regimen, valency, and affinity on brain uptake, TfR expression, and Fc-effector function side effects. These studies have primarily used monovalent TfRMAbs with a human constant region following acute intravenous dosing in mice. The effects of a high-affinity bivalent TfRMAb with a murine constant region, without a fusion partner, following extravascular dosing in mice are, however, not well characterized. Here we elucidate the plasma pharmacokinetics and safety of a high-affinity bivalent TfRMAb with a murine constant region following acute and chronic subcutaneous dosing in adult C57BL/6J male mice. Mice received a single (acute dosing) 3 mg/kg dose, or were treated for four weeks (chronic dosing). TfRMAb and control IgG1 significantly altered reticulocyte counts following acute and chronic dosing, while other hematologic parameters showed minimal change. Chronic TfRMAb dosing did not alter plasma- and brain-iron measurements, nor brain TfR levels, however, it significantly increased splenic-TfR and -iron. Plasma concentrations of TfRMAb were significantly lower in mice chronically treated with IgG1 or TfRMAb. Overall, no injection related reactions were observed in mice.

## 1. Introduction

Alzheimer’s disease (AD) is a progressive neurodegenerative disorder with a high prevalence, and an estimated 24 million people worldwide and 5.8 million people in America suffer from AD [1]. Currently, there is no FDA-approved disease-modifying treatment for AD, and the existing drugs for AD, which were FDA-approved more than a decade ago, only provide symptomatic relief. The largest class of disease-modifying therapies being developed for AD target amyloid-beta or tau, and the majority (>50%) of these drug candidates are biologics including anti-tau and anti-amyloid beta antibodies [2].

One of the major hurdles while trying to develop a biologic, including anti-amyloid and anti-tau therapies for AD, is low brain bioavailability of the biologic due to the blood-brain barrier (BBB) [3]. One approach to non-invasively deliver biologics into the brain across the BBB is to target endogenous BBB receptor-mediated transcytosis (RMT) systems, which exist for ligands, such as transferrin, leptin, insulin, and insulin-like growth factors [4]. Among the RMT systems, the transferrin receptor-1 (TfR) is the most extensively studied for its ability to deliver biologics into the brain [5]. Antibodies against the TfR (TfRMAbs) that target epitopes spatially removed from the Tf binding site on the TfR are under pre-clinical and clinical development for brain drug delivery [5,6,7,8,9,10,11].

The majority of the initial work showing the efficacy of TfRMAbs as drug delivery vehicles in the mouse has focused on the use of a rat/mouse chimeric high-affinity TfRMAb comprised of variable regions from a rat IgG fused to constant regions from mouse IgG1/kappa [12,13]. In the last decade, there has been an increased effort to engineer different variants of the TfRMAb and a review of the published studies highlights differences in the in vivo behavior of these TfRMAbs depending on several factors including: (1) valency of the antibody (monovalent versus bivalent), (2) presence or absence of a therapeutic fusion partner, (3) the species origin of the constant region, and/or (4) the dosing regimen (acute versus chronic and routes of administration) [8,9,11]. While bivalent TfR engagement results in high-affinity antibodies and efficacy at low doses (1–5 mg/kg) [10,12,13,14,15], monovalent TfR engagement results in low-affinity antibodies that are administered at higher doses (20–50 mg/kg) [8,9,11,16]. Differences in brain uptake are reported with these different TfRMAb variants. In mice, brain uptake and TfRMAb antibody affinity showed a direct correlation at low non-TfR-saturating doses, such that the high-affinity TfRMAb resulted in higher brain uptake [11]. However, at high-TfR-saturating doses, the low-affinity monovalent TfRMAb resulted in a more sustained brain exposure compared to the high-affinity variant [8,11], owing to selective saturation of the BBB TfR by high doses of the high-affinity TfRMAb in the mouse. In contrast, in non-human primates, the high-affinity variant resulted in a higher brain uptake and efficacy compared to the low-affinity variant even at high injection doses [16].

Apart from differences in brain drug delivery, differences are reported in the safety aspects of these TfRMAb variants. Previous studies using monovalent TfRMAbs with a humanized constant region, with and without a fusion partner, showed severe injection related reactions, including acute clinical signs and reticulocyte suppression, and increased cytokine release, after a single intravenous (IV) injection in mice [8,9]. Notably, no injection-related reactions were observed following subcutaneous (SQ) administration [17]. Further, studies with the high-affinity monovalent TfRMAb fused to a therapeutic antibody (anti-β-secretase (BACE1)) showed a dose-dependent reduction in BBB TfR levels in vivo and in vitro, which correlated with reduced brain uptake in mice [18]. A similar reduction in BBB TfR was reported in non-human primates [16]. Notably, these effects were not seen with a low-affinity TfRMAb-anti-BACE1 antibody fusion protein [16,18]. With respect to iron metabolism and hematologic safety, no significant long-lasting alterations were reported following chronic IV dosing of a monovalent low-affinity TfRMAb-anti-BACE1 fusion protein with a human constant region in mice [8]. The impact of a high-affinity TfRMAb on iron metabolism and hematologic indices following chronic dosing was not reported.

These safety data, including the behavior, hematologic- and iron-indices, of TfRMAbs largely focus on monovalent TfRMAbs with a humanized constant region, which has the potential to trigger an immune response, following acute and chronic IV dosing, in mice [8,9]. There are two safety studies of high-affinity bivalent TfRMAbs with a murine Fc-region fused to fusion partners, following chronic IV and SQ dosing [10,19]. There is only one study that reports the safety data of a humanized high-affinity bivalent TfRMAb, not fused to a fusion partner, in non-human primates following chronic IV dosing [7]. If a TfRMAb is to be used as a brain drug delivery vector for a chronic neurodegenerative disease like AD, the SQ route will be more optimal than the IV route for chronic dosing [17], and there are no studies that report the comprehensive safety profile of a high-affinity bivalent TfRMAb, without a fusion partner, following acute and chronic SQ dosing, in mice. This study aims to fill this gap by characterizing the effect of a high-affinity bivalent TfRMAb, with a murine constant region, on hematologic-, iron- and behavior-indices following SQ acute- and chronic-dosing at low therapeutic doses, in mice.

## 2. Materials and Methods

### 2.1. Antibody Production and Purification

ExpiCHO cells grown in serum-free expression medium (Gibco, Gaithersburg, MD, USA), were used to produce the TfRMAb (Genscript, Piscataway, NJ, USA). TfRMAb was purified with a protein G column and size exclusion chromatography (SEC). Sodium dodecyl sulfate polyacrylamide gel electrophoresis (SDS-PAGE), SEC HPLC, and endotoxin were assessed for final TfRMAb assessment and the protein was stored at −80 °C formulated in 10 mM sodium acetate, 150 mM NaCl (Thermo Fisher Scientific, Waltham, MA, USA) 0.01% polysorbate 80(Thermo Fisher Scientific, Waltham, MA, USA), pH = 6 at a concentration of 1.05 mg/mL. The TfRMAb is derived from the heavy and light chain variable regions of the 8D3 MAb against the mouse TfR, and the mouse IgG1 and kappa heavy and light chain constant regions [13].

### 2.2. Acute Dosing of Transferrin Receptor Monoclonal Antibody

**Figure 1:**
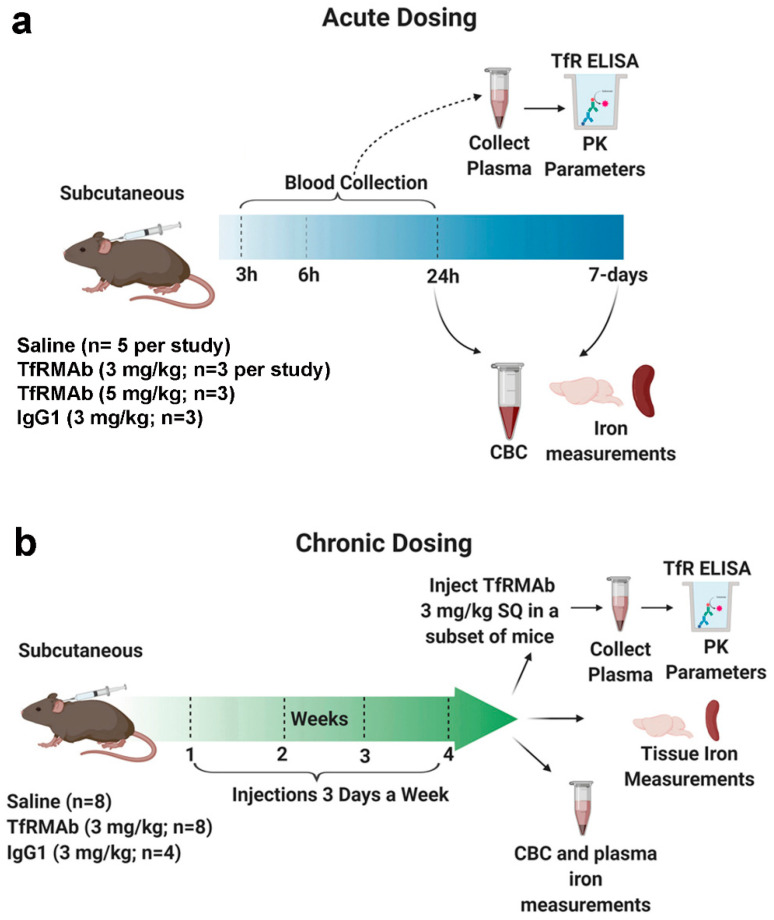
Schematic diagram summarizing the experimental study design for acute (**a**) and chronic (**b**) dosing. For acute dosing study, 22 mice were randomly assigned to receive 3 mg/kg (*n* = 3) or 5 mg/kg (*n* = 3) SQ dose of TfRMAb, 3 mg/kg SQ IgG1 control (*n* = 3), or saline (*n* = 5). Plasma was collected at 3-, 6-, and 24-h after injection to determine TfRMAb plasma concentrations in mice dosed with the 3 and 5 mg/kg TfRMAb. Terminal whole blood was collected for a complete blood count (CBC), and brains and spleens were excised. No CBC was performed on the 5 mg/kg dose. For the seven-day study, mice were treated with a single 3 mg/kg SQ TfRMAb dose (*n* = 3) or saline (*n* = 5) and observed for seven days after which whole blood was collected for a CBC, and brains were excised (**a**). For chronic dosing study, twenty mice were randomly assigned to receive saline (*n* = 8), 3 mg/kg TfRMAb (*n* = 8) or 3 mg/kg IgG1 (*n* = 4) SQ three days a week for four weeks. Blood was collected at the end of two weeks for a CBC and at the end of four weeks for iron parameters. A plasma PK study was performed one week after the end of chronic dosing and mice from all the experimental groups received a 3 mg/kg SQ dose of TfRMAb (**b**). The figure was created using Biorender.com.

All the animal studies were performed on eight-week old male C57BL/6J mice (Jackson Laboratory, Bar Harbor, ME, USA) under protocols approved by the University of La Verne Institutional Animal Care and Use Committee (LV0012c, 2 October 2019). Mice were provided constant access to food and water and maintained on a 12 h light/12 h dark cycle. To simulate acute treatment, the mice were given a single injection of either saline (Teknova, Hollister, CA, USA) (*n* = 5) or the high-affinity bivalent TfRMAb (3 mg/kg; *n* = 3 or 5 mg/kg; *n* = 3), via the SQ route (Figure 1). Mice treated with mouse IgG1 (3 mg/kg SQ, Clone: MOPC-21, Bio X Cell, West Lebanon, NH, USA; *n* = 3) served as additional controls [13]. Blood was collected in sodium citrate (Thermo Fisher Scientific, Waltham, MA, USA) at 3 h, 6 h, and 24 h after injection, after which cardiac perfusion was performed. Mice were euthanized, and brains and spleens were harvested. The blood was spun down to collect the plasma as described previously to determine plasma TfRMAb concentrations [20]. An additional terminal whole blood aliquot was taken for a complete blood count (CBC) (Molecular Diagnostic Services, Inc., San Diego, CA, USA) [20]. The overall health of the mice was evaluated immediately and 24-h after injection to record any abnormal clinical signs by assessing the following parameters: general appearance, posture, body conditions, respiration, body weight, spontaneous locomotion/social interaction, and urine color [21].

To determine if the effects of acute dosing of the TfRMAb are reversible or long-lasting, a separate group of mice was sacrificed at seven days after the single injection mentioned above. For this, mice received either saline (*n* = 5) or 3 mg/kg (*n* = 3) TfRMAb via the SQ route. Seven days after the single injection, terminal blood was collected for a CBC, cardiac perfusion was performed, mice were euthanized, and brains were harvested.

### 2.3. Chronic Dosing of Transferrin Receptor Monoclonal Antibody

To mimic treatment needed for chronic neurodegenerative diseases including AD, a chronic dosing study was performed (Figure 1). For this, the mice were injected SQ three times a week for four weeks with either saline (*n* = 8), 3 mg/kg IgG1 (*n* = 4), or 3 mg/kg TfRMAb (*n* = 8). Saline mice received an equivalent volume of saline. The overall health of the mice was evaluated immediately after injection and weekly as described below. Blood was collected for a CBC (*n* = 3 per group) at the end of two weeks. At the end of four weeks an aliquot of plasma was used to measure iron parameters (*n* = 4 per group). Iron parameters were not measured for the mouse IgG1-treated mice. For CBC, blood was collected in sodium citrate whereas for iron parameters blood was collected in heparinized vials (Thermo Fisher Scientific, Waltham, MA, USA). To determine the impact of chronic TfRMAb dosing on TfRMAb plasma PK, mice chronically treated with saline, IgG1 or TfRMAb received a single 3 mg/kg dose of the TfRMAb via the SQ route one week after chronic four-week dosing. Blood was collected at 3 h, 6 h, and 24 h after the injection, and the separated plasma was used for an ELISA to determine plasma concentrations of the TfRMAb in the mice that were chronically treated with either saline (*n* = 4), IgG1 (*n* = 4) or TfRMAb (*n* = 4). After the blood collection, cardiac perfusion was performed, mice were euthanized, and brains and spleens were harvested.

### 2.4. ELISA for Plasma Antibody Concentrations

Plasma TfRMAb concentrations were determined using a TfR ELISA as described previously with slight modifications [20]. Mouse TfR (200 ng/well, R&D system, Minneapolis, MN, USA) constituted in 0.05 M NaHCO_3_ (Sigma-Aldrich, St. Louis, MO, USA) at pH 8.3 was used as the coating agent to coat Nunc Maxisorp plates (Fisher Scientific, Waltham, MA, USA) overnight at 4 °C. Tris-buffered saline (TBS, Sigma-Aldrich, St. Louis, MO, USA) containing 0.05% Tween-20 (TBSB) was used as the blocking agent. Wells were incubated with TfRMAb standards and diluted plasma (1:10 in TBSB) for 1 h at room temperature followed by incubation with the alkaline phosphatase conjugated goat anti-mouse light chain (kappa) secondary antibody (Bethyl Laboratories Inc., Montgomery, TX, USA) for 45 min at room temperature. P-nitrophenyl phosphate (Sigma-Aldrich, St. Louis, MO, USA) was used as the substrate and the reaction was stopped by adding 1.2 M NaOH (Sigma-Aldrich, St. Louis, MO, USA). Absorbance (OD) was measured at 405 nm and OD was corrected with TBSB-blank values. Standard curves were used to determine maximum binding (B_max_) and dissociation constant (K_d_) (GraphPad Prism 8, San Diego, CA, USA) as described previously [20].

### 2.5. Tissue Iron Measurements

Iron levels in brain and spleen samples were measured by Agilent 8900 triple quadrupole Inductively Coupled Plasma Mass Spectrometry (ICP-MS, Santa Clara, CA, USA). Briefly, 5 µL of 67% nitric acid (Thermo Fisher Scientific, Waltham, MA, USA) per mg brain sample was used to lyse the tissue overnight at room temperature, followed by a 1 h digestion at 90 °C using 5 µL of 30% hydrogen peroxide (Sigma-Aldrich, St. Louis, MO, USA) per mg brain tissue. For spleen samples, 10 µL of 67% nitric acid(Sigma-Aldrich, St. Louis, MO, USA) per mg spleen sample was used for lysis overnight at room temperature, followed by a 1 h digestion at 90 °C using 10 µL of 30% hydrogen peroxide per mg spleen tissue. Both brain and spleen samples were further diluted (1:5) with 1% nitric acid before iron detection by ICP-MS. Calibration curves were drawn from calibration blanks at six standard points with different iron concentrations (4 nM–4 µM). For the acute dosing study, brain iron levels were determined in the saline (*n* = 3) and TfRMAb (*n* = 3) treated mice. Spleen iron levels were not determined following acute dosing. For the chronic dosing study, brain and spleen iron levels were determined in saline (*n* = 4) and TfRMAb (*n* = 4) treated mice. Brain and spleen iron levels were not determined in the mouse IgG1 treated mice.

### 2.6. Cell Culture

Murine brain microvascular endothelial cells (bEND.3 cells; American Type Culture Collection, Manassas, VA, USA) were maintained in Dulbecco’s Modified Eagle’s Medium (DMEM, ATCC, Manassas, Virginia, USA) supplemented with 10% fetal bovine serum (Thermo Fisher Scientific Waltham, MA, USA), and 100 μg/mL penicillin/streptomycin (Sigma, St. Louis, MO, USA) at standard cell culture conditions (37 °C, 5% CO_2_, 95% air). Cells used were between passages 22 and 31 and were seeded onto 96- (Corning, New York, NY, USA) and six-well plates (Thermo Fisher Scientific, Waltham, MA, USA).

### 2.7. Western Blot for Transferrin Receptor (TfR) and Cell Viability

To detect TfR protein levels in brain endothelial cells, bEND.3 cells grown in six-well plate were treated with TfRMAb followed by lysis in 50 µL radioimmunoprecipitation assay (RIPA) buffer (Thermo Fisher, Waltham, MA, USA) with cocktail protease inhibitors (Bio-rad, Hercules, CA, USA) per well. TfR protein levels were determined in a subset of brain and spleen tissue samples collected from saline-, TfRMAb- and IgG1-treated mice after acute (*n* = 3 per group) and chronic dosing (saline *n* = 7 for brain samples and *n* = 8 for spleen samples, TfRMAb *n* = 4, IgG1 *n* = 3 for brain samples and *n* = 4 for spleen samples). To detect TfR protein levels in brain and spleen tissue samples, 100 µL RIPA buffer with cocktail protease inhibitors per mg tissue sample was used for lysis. Samples were homogenized in lysis buffer followed by a 1 h incubation on ice. Samples were centrifuged at 12,000× *g* for 20 min at 4 °C and the supernatant was collected, and boiled in Laemmli buffer (Bio-rad, Hercules, CA, USA) containing 10% 2-mercaptoethanol (Bio-rad, Hercules, CA, USA) for Western blot. Briefly, blots were probed with a non-TfRMAb-competing anti-transferrin receptor antibody (1:1000 dilution; Invitrogen, cat. # 136800, Carlsbad, CA, USA) at 4 °C overnight. Membranes were then exposed to the appropriate horseradish peroxidase–conjugated secondary antibody (1:10,000 dilution; Invitrogen, cat. #G-21040, Carlsbad, CA, USA), followed by chemiluminescence detection (Thermo Fisher Scientific, Waltham, MA, USA). Equal protein loading was controlled by probing the membrane with an anti-β-actin antibody (1:1000 dilution; Santa Cruz Biotechnology, Dallas, TX, USA). Chemiluminescence was detected using the UVP ChemiDoc-It TS2 Imager (Upland, CA, USA), and the intensity of the Western blot bands was analyzed using NIH ImageJ (version 1.52a, Bethesda, MD, USA) as follows. The largest western blot band was selected using the rectangular tool in NIH ImageJ and densitometry measurements were performed for each lane/band keeping the box size the same. Band intensity was normalized to the β-actin band intensity. The Western blot data was reported as % of control.

### 2.8. Cell Viability

bEND.3 cells were seeded in 96-well plates (Corning, New York, NY, USA) at density of 7500 cells/well. Following a 24–72 h incubation with TfRMAb at concentrations of 0.2, 2, 20, 200, and 2000 nM, cell viability was measured by the Cell Counting Kit-8 (CCK-8) assay (Dojindo Molecular Technologies, Rockville, MD, USA). Briefly, 10 µL of CCK-8 solution was added into each well-containing cells, and cells were incubated for 3 h at 37 °C. The absorbance at 450 nm was measured using a plate reader and cell viability was normalized to % of the bEND.3 control group.

### 2.9. Pharmacokinetic Analysis

Non-compartmental analysis to calculate PK parameters (maximum plasma concentration (Cmax), area under the plasma concentration-time curve (AUC), time to reach maximum plasma concentration (Tmax), and clearance/bioavailability (apparent clearance)) was performed using Kinetica 5.0 (Thermo Fisher Scientific, Waltham, MA, USA). The following PK parameters were compared: Cmax ratio, AUC ratio and clearance ratio as follows. To highlight the relative changes in PK parameters between the 3 and 5 mg/kg doses, the 3 mg/kg AUC, Cmax and clearance values were normalized to the 5 mg/kg dose to give the AUC ratio, Cmax ratio and clearance ratio, respectively. To highlight relative changes from the saline-treated group in the chronic dosing PK study, AUC, Cmax and clearance values were normalized to saline + TfRMAb treated mice values to give the AUC ratio, Cmax ratio and clearance ratio.

### 2.10. Statistical Analysis

Statistical analysis was performed using GraphPad Prism 8 (GraphPad Software Inc., LA Jolla, CA, USA) and all data is presented as the mean ± SEM. Power analysis was performed using Minitab (Minitab LLC, State College, PA, USA) to estimate the sample size, which showed that a sample size of 3–4 mice per group will be sufficient to detect a predicted effect size (between 40% and 50%), with a standard deviation of 10–15%, 80% power and significance level of 5%, based on previous similar studies [20]. An independent two-sample *t* test or one-way ANOVA with Holms–Sidak post-test was used to compare two or more than two groups, respectively. Cmax, AUC and clearance ratios were compared to a hypothesized value = 1, indicating no change, using one-sample t-test. A two-tailed *p-*value of less than 0.05 was considered statistically significant.

## 3. Results

### 3.1. Plasma Pharmacokinetics after Acute Dosing

**Figure 2:**
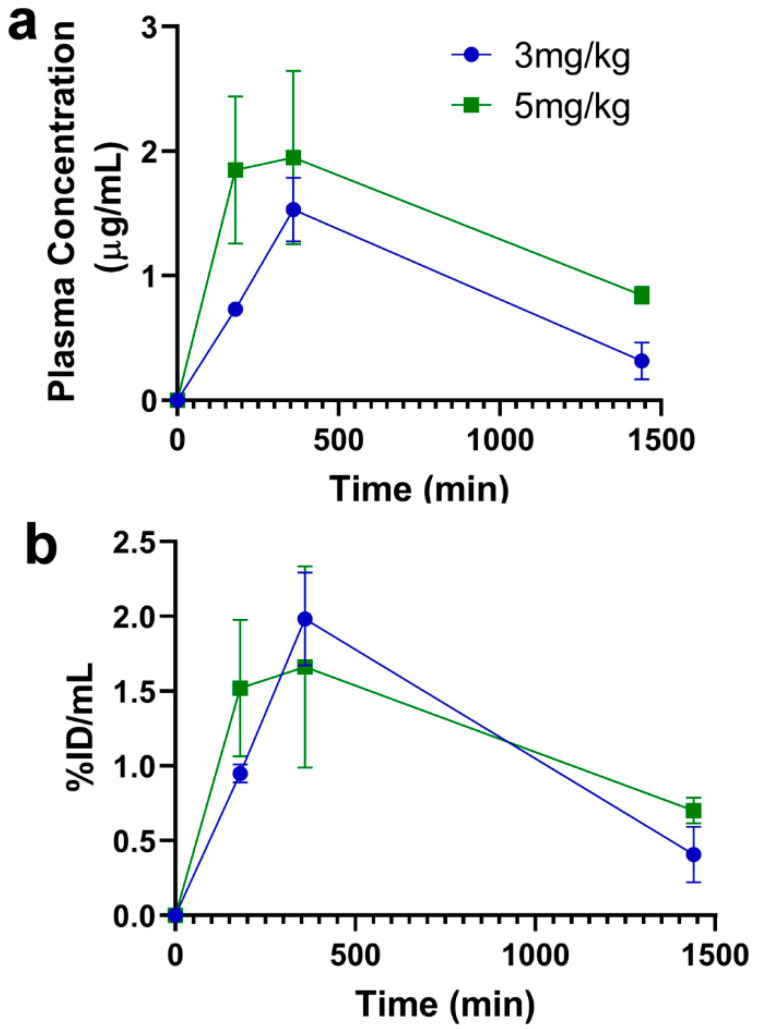
Plasma concentration-time profiles of the high-affinity bivalent TfRMAb following SQ injection. Mice were injected with TfRMAb (3 mg/kg or 5 mg/kg) via the SQ route and plasma samples were collected at 3 h (180 min), 6 h (360 min), and 24 h (1440 min) following injection. Plasma concentrations are shown as either µg/mL (**a**) or %ID/mL (**b**). Data are shown as the mean ± SEM of *n* = 3 per dose per time point.

TfRMAb plasma concentrations in µg/mL and % injected dose (% ID)/mL following 3 mg/kg and 5 mg/kg SQ injections, are shown in Figure 2. The Cmax was 1.5 ± 0.3 µg/mL (1.9 ± 0.4% ID/mL) and 2.3 ± 0.7 µg/mL (1.7 ± 0.4% ID/mL) for 3 mg/kg and 5 mg/kg dose, respectively. There was a trend towards a change in Cmax (*p* = 0.07) and AUC (0–24 h) (*p* = 0.08) with a change in dose from 3 mg/kg to 5 mg/kg (Table 1). The 3 mg/kg dose is 40% lower than the 5 mg/kg dose and accordingly, the Cmax with a 3 mg/kg dose was ~34% lower, while the AUC (0–24 h) was ~37% lower compared with the 5 mg/kg dose. The plasma Tmax was 360 min for both the doses (Table 1).

### 3.2. Complete Blood Count (CBC) after Acute and Chronic Dosing

**Figure 3:**
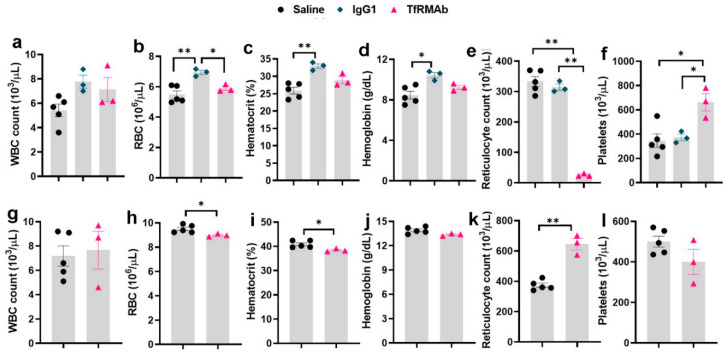
Complete blood count (CBC) data following acute dosing of the high-affinity bivalent TfRMAb in mice. Mice were treated with a 3 mg/kg SQ dose of TfRMAb, IgG1, or an equivalent volume of saline, and a CBC was performed either 24 h (**a**–**f**) or seven days (**g**–**l**) after the injection. No CBC data was collected for the mouse IgG1 treated mice at seven days. Data area shown as the mean ± SEM of *n* = 3–5 per group. * *p* < 0.05, ** *p* < 0.01.

CBC data following acute dosing is shown in Figure 3. Within 24 h following a single 3 mg/kg SQ dose, there was a significant increase in RBC-indices (26% increase in RBC, 27% increase in hematocrit, and 23% increase in hemoglobin; Figure 3b–d) in the IgG1 treated mice compared to saline-controls. No change in these parameters was observed in the TfRMAb treated mice. Reticulocytes were significantly reduced in mice treated with TfRMAb (92% reduction) (Figure 3e). Platelets were significantly elevated (91% increase) in the TfRMAb treated mice within 24 h following a single injection (Figure 3f). Seven days after the single injection, platelets normalized to control values while the reticulocytes were significantly elevated (72% increase) in the TfRMAb treated mice compared to saline-controls (Figure 3k,l). There was a small but significant reduction in RBC (5% reduction) and hematocrit (6% reduction) seven days after a single TfRMAb injection (Figure 3h,i).

**Figure 4:**
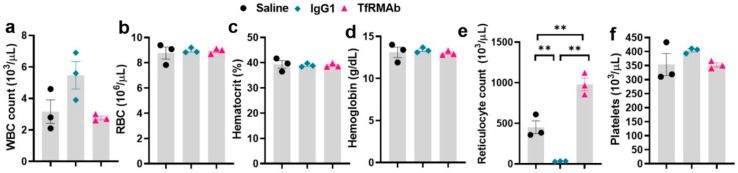
Complete blood count (CBC) data following chronic dosing of the high-affinity bivalent TfRMAb in mice. Mice were treated 3 days per week for four-weeks with a 3 mg/kg SQ dose of TfRMAb, IgG1, or an equivalent volume of saline, and a CBC (**a**–**f**) was performed at the end of two weeks. Data are shown as the mean ± SEM of *n* = 3 per group. ** *p* < 0.01.

CBC data following chronic dosing is shown in Figure 4. While there was a significant increase (118% increase) in reticulocytes following chronic TfRMAb dosing, reticulocyte reduction (92% decrease) was observed following chronic IgG1 dosing (Figure 4e). All other parameters (WBC, RBC, hemoglobin, hematocrit, and platelets) remained unchanged following chronic dosing (Figure 4a–d,f). The RBC indices (RBC number, hematocrit, and hemoglobin) are lower for the 24-h study (Figure 3b–d) compared with the seven-day study (Figure 3h–j) and chronic dosing study (Figure 4b–d), which can be attributed to the acute effect of blood collection at 3, 6, and 24 h during the 24-h study.

### 3.3. Iron Measurements

**Figure 5:**
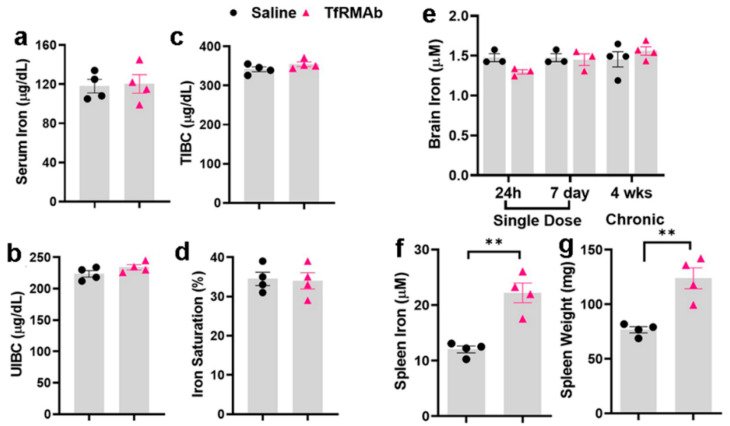
Plasma and organ iron-measurements following acute and chronic dosing of the high-affinity bivalent TfRMAb in mice. Mice were treated three days per week for four weeks with a 3 mg/kg SQ dose of TfRMAb, and plasma iron-indices were measured (**a**–**d**). Whole-brain tissue iron at 24 h and seven days after acute dosing and four-weeks after chronic TfRMAb dosing (**e**). Spleen-iron and -weights four weeks after chronic TfRMAb dosing (**f**,**g**). Data are shown as the mean ± SEM of *n* = 3–4 per group. ** *p* < 0.01. TIBC: Total-iron binding capacity; UIBC: unsaturated iron-binding capacity.

The effect of chronic TfRMAb dosing on plasma and tissue iron levels is shown in Figure 5. All the plasma iron-indices—serum iron (Figure 5a), unsaturated iron-binding capacity (UIBC; Figure 5b), total-iron binding capacity (TIBC; Figure 5c), and iron saturation (Figure 5d)—remained unchanged following chronic TfRMAb dosing. Similarly, no change in whole brain tissue iron was observed following acute and chronic TfRMAb dosing (Figure 5e). Chronic TfRMAb dosing resulted in a significant (83%) increase in splenic-iron (Figure 5f) and significant splenic-enlargement (62% increase) (Figure 5g). No change in brain or kidney weights or total body weights of mice was observed following chronic dosing (data not shown).

### 3.4. TfR Levels In Vivo and In Vitro

**Figure 6:**
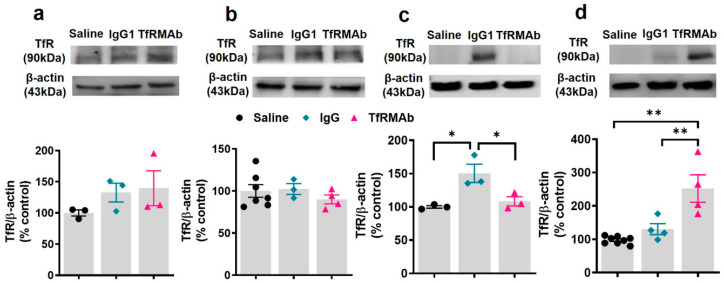
Transferrin receptor (TfR) protein levels following acute and chronic dosing of the high-affinity bivalent TfRMAb in mice. Mice were treated with a single 3 mg/kg SQ dose or three days per week for four weeks with a 3 mg/kg SQ dose of TfRMAb, and brain (**a**,**b**) and spleen (**c**,**d**) TfR protein levels were measured. Whole brain tissue TfR protein after 24 h (**a**) and chronic (**b**) TfRMAb dosing. Spleen TfR protein after 24 h (**c**) and chronic (**d**) TfRMAb dosing. TfR levels were detected by Western blotting using a non-competing antibody against the TfR. Data are shown as the mean ± SEM of *n* = 3–8 per group. * *p* < 0.05, ** *p* < 0.01.

The effect of acute (single injection) and chronic dosing of TfRMAb on TfR protein levels in vivo is shown in Figure 6. Acute and chronic four-week treatment with the TfRMAb did not significantly alter brain TfR protein levels (Figure 6a,b) detected by Western blotting using a TfR antibody recognizing an epitope distinct from the TfRMAb binding domain. Acute dosing of the TfRMAb did not affect the splenic TfR protein levels. Acute IgG1 dosing on the other hand resulted in a significant increase in the splenic TfR protein compared to saline controls (51% increase) and TfRMAb-treated (39% increase) mice at 24 h after treatment (Figure 6c). Following chronic dosing, we observed a significant increase in the splenic TfR protein levels in the TfRMAb treated mice compared to saline- (157% increase) and IgG1-treated (94% increase) mice (Figure 6d).

**Figure 7:**
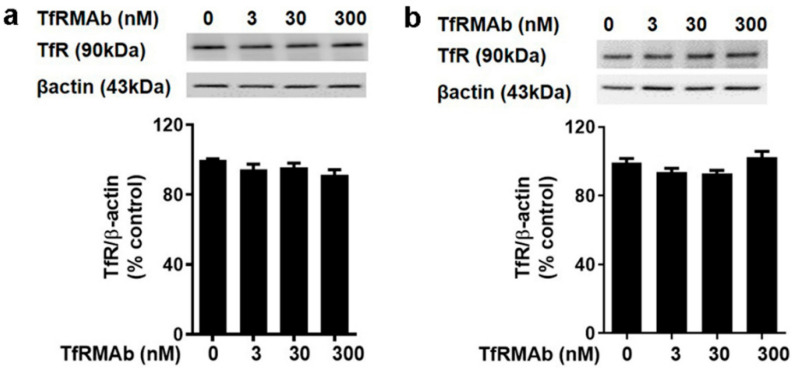
TfR protein levels in cultured mouse brain endothelial cells following acute and chronic treatment with the high-affinity bivalent TfRMAb. bEND.3 cells were treated with indicated doses of TfRMAb for 24 h (**a**) or 96 h (**b**). TfR levels were detected by western blotting using a non-competing antibody against the TfR. Data is shown as the mean ± SEM of *n* = 3 independent experiments done in duplicates.

The effect of acute and chronic TfRMAb treatment on cultured mouse brain endothelial cells (bEND.3 cells) is shown in Figure 7. Treatment of bEND.3 cells with TfRMAb for up to 24 h (to mimic acute dosing) (Figure 7a) and 96 h (to mimic chronic dosing) (Figure 7b) did not alter TfR protein levels for TfRMAb concentrations ranging from 0 to 300 nM. No change in brain endothelial cell viability was observed when cells were treated with 0–200 nM TfRMAb for up to 24 h and 48 h (data not shown). There was a significant decrease in brain endothelial cell viability at high concentrations (2000 nM) of the TfRMAb (data not shown).

### 3.5. Plasma Pharmacokinetics after Chronic Treatment

**Figure 8:**
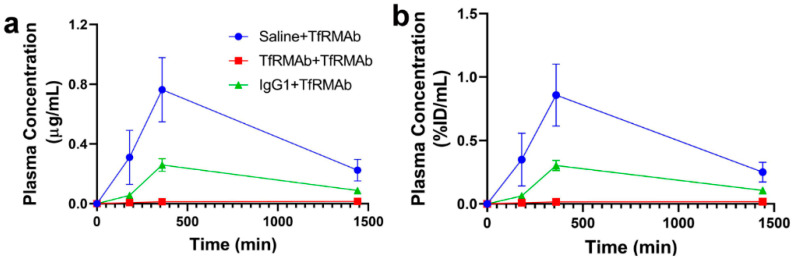
Plasma concentration-time profiles of the high-affinity bivalent TfRMAb following chronic dosing in mice. Mice were treated with a single 3 mg/kg TfRMAb SQ injection one week after chronic four-week treatment with saline, TfRMAb or IgG1 (*n* = 4 per group), and plasma samples were collected at 3 (180 min), 6 (360 min) and 24 (1440 min) hours after injection. Plasma TfRMAb concentrations are shown as either µg/mL (**a**) or %ID/mL (**b**). Data is shown as the mean ± SEM.

Plasma concentrations of TfRMAb in mice treated with a single 3 mg/kg TfRMAb SQ injection after chronic four-week treatment with saline, TfRMAb, or IgG1 are shown in Figure 8 and are represented in µg/mL (a) and % ID/mL (b). The Cmax of TfRMAb was 0.76 ± 0.2 µg/mL (0.86 ± 0.24% ID/mL), 0.016 ± 0.007 µg/mL (0.019 ± 0.008% ID/mL) and 0.26 ± 0.04 µg/mL (0.3 ± 0.04% ID/mL) in the saline-treated, TfRMAb-treated and IgG1-treated mice, respectively. In the mice that were chronically dosed with TfRMAb and IgG1, the AUC (0–24) of TfRMAb was 97% lower (*p* < 0.01) and 66% lower (*p* < 0.01), respectively, than that in the mice chronically dosed with saline (Table 2). The apparent clearance of TfRMAb in mice chronically dosed with IgG1 or TfRMAb was 130% (*p* < 0.05) and 5300% (*p* = 0.09) higher, respectively, than the clearance of TfRMAb in mice chronically treated with saline (Table 2).

The overall well-being of mice following acute and chronic dosing was evaluated to see if any adverse events were present after injections. All the parameters assessed remained unaltered and no injection reactions or clinical signs were observed following acute or chronic dosing in both the TfRMAb and IgG1 treated mice (data not shown).

## 4. Discussion

In the current study, we elucidate the impact of a high-affinity bivalent TfRMAb with a murine IgG1/kappa constant region on hematologic-, iron-, and plasma PK-indices, and overall health following acute and chronic SQ dosing in mice. Following acute treatment, TfRMAb results in reticulocyte suppression, which is reversed within seven days, and no reticulocyte suppression is seen following chronic TfRMAb dosing. Both acute and chronic TfRMAb dosing had no major long-lasting impact on any other hematologic parameter measured in the current study. Plasma and brain iron measurements remained stable with TfRMAb treatment. However, a significant increase in splenic-iron was observed with chronic TfRMAb dosing, which was associated with a significant increase in splenic-TfR protein levels. TfRMAb treatment did not alter brain TfR protein levels in the current study. Chronic antibody dosing significantly altered the plasma PK of TfRMAb and there was a decrease in the plasma concentrations of TfRMAb following chronic IgG1 or TfRMAb dosing. No injection related reactions were observed during acute and chronic dosing studies.

Given the ease of administration and reduced potential for adverse effects associated with SQ antibody dosing [17], the current studies focused on SQ dosing. Following a single SQ injection (acute dosing), an increase in dose from 3 mg/kg to 5 mg/kg resulted in a proportional increase in plasma concentrations and AUC, though this increase did not reach statistical significance and only showed a trend (Figure 2 and Table 1). Since majority of our previous work with TfRMAb-based fusion proteins was done using a 3 mg/kg dose [10,22,23], we focused on this dose in the current study. Following acute dosing, TfRMAb-treatment resulted in significant reticulocyte suppression (Figure 3). The reduction in reticulocytes following TfRMAb treatment is consistent with previous work with TfRMAbs, with and without therapeutic fusion partners [8,20], and is shown to be mediated in part via the reticulocyte TfR-TfRMAb interaction, antibody-dependent cell-mediated cytotoxicity and the complement cascade [8]. We observed a significant increase in platelets in the TfRMAb treated mice at 24 h after a single injection. This increase in platelets, which has not been previously reported with TfRMAb treatment, was an acute and reversible response that normalized within seven days, and the implication of this finding needs further investigation. Acute single-dose treatment of the high-affinity TfRMAb did not alter the other hematologic indices (RBC, hematocrit, and hemoglobin), which is consistent with previous reports using TfRMAbs with humanized constant region in mice [8].

To determine if the acute effects of TfRMAb were long-lasting, we performed a CBC at seven days after the single SQ injection. Reticulocyte-suppression that was observed 24 h following a single TfRMAb SQ dose, was reversed seven days following acute dosing, and reticulocytes were significantly elevated seven days after treatment. This is consistent with a previous report showing an increase in reticulocytes seven days after a single injection of TfRMAb fused to a therapeutic fusion partner [8]. Reticulocyte suppression seen with the high-affinity TfRMAb is, therefore, an acute but reversible response. An increase in circulating reticulocytes has been observed following blood loss, hemolysis, or erythropoietin administration [24], and the increase in reticulocytes seen seven days after the single injection in the current study may be a compensatory stress response associated with reduced reticulocytes seen 24 h after TfRMAb dosing. We also observed a small (3%) but a significant decrease in hemoglobin levels and a trend towards a decrease in RBC in mice treated with TfRMAb seven days after the single injection. This decrease may be a response to the reduced reticulocytes seen 24 h after injection.

Since AD is a neurological disease that requires chronic treatment, we performed a chronic-dosing four-week study to determine the impact of continuous TfRMAb treatment on CBC indices, brain TfR levels, plasma and brain iron levels, plasma PK of TfRMAb, and overall health of the mice. Following chronic SQ dosing, no changes in CBC indices were observed showing that any effects of TfRMAb on CBC indices seen during the single dosing study were acute and not seen with long-term treatment (Figure 4). The only significant change following chronic TfRMAb dosing was a significant elevation in reticulocytes, which is consistent with our previous work showing an increase in reticulocytes following chronic TfRMAb-EPO fusion protein dosing at four-weeks [10]. This increase in reticulocytes with the TfRMAb-EPO fusion protein normalized by eight weeks of treatment. Whether the increase in reticulocytes seen with the TfRMAb without a fusion partner in the current study returns to normal with longer treatment duration, needs further investigation. Interestingly, chronic dosing with the control antibody, the MOPC21 mouse IgG1, resulted in a profound reduction in reticulocytes in the current study and this was an unexpected finding. It should be noted that the clearance of the TfRMAb is expected to be higher than the control IgG1 given the TfR-mediated peripheral clearance of the TfRMAb. Therefore, the plasma concentrations of the control IgG1 are expected to be much higher than those of TfRMAb for the same dosing regimen and the effects of the TfRMAb and IgG1 cannot be directly correlated. The significant reticulocyte reduction seen in the IgG1 treated mice, suggests that reduction in reticulocytes may be TfRMAb independent. This reticulocyte reduction seen by chronic IgG1 dosing may be mediated via other mechanisms, such as reticulocyte opsonization and subsequent clearance by splenic and liver macrophages activated by the complement system, as suggested previously [8]. It is interesting to note that high dose immunoglobulin treatment is associated with anemia [25] and reticulocytopenia in some cases [26]. Future long-term studies will be needed to determine if the reduction in reticulocytes observed with chronic IgG1 dosing in the current study translates into reduced RBC counts at a later time, resulting in anemia.

Throughout the acute and chronic dosing study, we did not see any clinical signs or compromise in the overall health of the mice (data not shown). This finding is consistent with recent work with a high-affinity bivalent humanized TfRMAb (3–30 mg/kg dose) without a therapeutic fusion partner in non-human primates [7], and our work with the high-affinity TfRMAb with a murine IgG1/kappa constant domain fused to a therapeutic protein (3 mg/kg dose) in mice [10]. These findings, however, differ from the severe acute clinical signs observed with monovalent TfRMAbs with a human constant domain for doses ranging from 1 to 50 mg/kg following IV injection in mice [8]. Our initial explanation for this difference was the use of humanized antibodies (IgG1) in mice [8]. However, a recent study showed similar severe acute clinical signs, including death, in mice treated with TfRMAb decorated liposomes [27]. The TfRMAb used in the above study was a rat IgG2 (TfRMAb clone RI7-217) that was injected IV. Based on this, one possible explanation for such marked differences in the presence of acute clinical signs with some TfRMAbs [8,27] but not with the TfRMAb used in the current study may be the isotype of the constant region of the TfRMAbs injected in mice and the ensuing Fc-effector function. Though different IgG isotypes are highly conserved, they differ in their constant regions and, hence, in their ability to induce effector function. For example, among the mouse IgG isotypes, mouse IgG1 results in lower effector function compared with mouse IgG2 that has a high binding affinity to the Fc-gamma receptor (FcγR), while human IgG1 has a high binding affinity to the FcγR and results in robust effector function [28]. Notably, the severe acute clinical signs reported with monovalent TfRMAbs with the human constant domain were attributed to Fc-mediated antibody-dependent cell-mediated cytotoxicity [8]. It is, therefore, conceivable that different isotypes of the IgG constant regions result in varying degree of effector function and resultant clinical signs following TfRMAb administration. Another difference between the published studies and the current study is the route of administration. Prior work showing severe acute clinical signs following TfRMAb dosing involved the IV route of administration [8,27], and a recent study showed that humanized-TfRMAbs result in first injection reactions following IV injection, but not following SQ dosing in mice [17]. One aspect that also needs further investigation is the impact of IgG constant region glycan pattern on the resultant effector function. It is known that IgGs produced in CHO cells have different glycan patterns (e.g., high fucose) compared with IgGs produced in myeloma cells, and afucosylated IgGs are potent inducers of effector function [29,30]. Notably, TfRMAb clone RI7-217 is produced in myeloma cells while the high-affinity TfRMAb used herein is produced in CHO cells. Future work will be needed to determine the impact of TfRMAb isotype, Fc-glycan pattern and route of administration on varied TfRMAb clinical responses.

Given the important role of the TfR in maintaining iron homeostasis [31], we also evaluated whether TfRMAb dosing affects peripheral and brain iron levels. The TfRMAb used in the current study has a high TfR binding affinity and is readily taken up by and transported across the brain endothelium [13]. However, the effect of this TfR-TfRMAb association on iron parameters and TfR levels is not reported. Our results show that chronic TfRMAb dosing does not alter plasma iron levels and iron-binding capacity, and does not change brain iron levels following acute and chronic TfRMAb dosing (Figure 5). These results together show that TfRMAb treatment does not alter brain iron homeostasis, and are consistent with previous work showing no changes in iron indices with monovalent TfRMAbs with a humanized constant region and bivalent TfRMAbs with a murine constant region in mice [8,19]. We, however, did observe a significant increase in splenic iron in mice treated with the TfRMAb for four weeks, which was associated with an enlargement of the spleen, changes that are consistent with splenic-iron-overload. To further examine if the changes in iron levels correlated with TfR levels, we measured TfR protein levels in the brains and spleens of mice treated with the TfRMAb (Figure 6 and Figure 7). We did not observe any change in the whole brain and brain endothelial TfR protein levels following acute and chronic TfRMAb treatments, which is consistent with no changes seen in brain iron levels with TfRMAb dosing. A significant reduction in mouse brain TfR protein levels has been reported with high-affinity TfRMAb doses between 25 and 50 mg/kg and not at lower doses (5 mg/kg) [18], which are comparable to the dose used in the current study. Similarly, in non-human primates, TfR reduction is observed with a 50 mg/kg dose and not with a 30 mg/kg dose of a high-affinity TfRMAb [16]. In contrast, low-affinity TfRMAbs do not affect brain TfR protein levels in mice or non-human primates [16,18]. Taken together, the impact of TfRMAbs on brain TfR protein levels is affinity- and, therefore, dose-dependent. High-affinity TfRMAbs, overall, do not alter brain TfR protein levels at low doses (3–5 mg/kg), and our results support this. Notably, high-affinity TfRMAbs are administered at low doses (1–5 mg/kg) given their high-affinity binding to the TfR, as opposed to low-affinity TfRMAbs that require high injection doses. In the current study, the highest plasma concentration of the high-affinity bivalent TfRMAb is < 17 nM (Cmax following SQ injection of 5 mg/kg dose is 2.3 ± 0.7 µg/mL; Table 1), and no change in brain endothelial TfR protein or cell viability was observed at this concentration in vitro. We, however, found a significant increase in splenic TfR protein levels following chronic TfRMAb dosing but not after acute treatment. This increase in splenic TfR may explain the increase in splenic iron and enlargement in TfRMAb-treated mice. We also found a significant increase in splenic TfR levels in mice treated with a single injection of IgG1. These results suggest that the increase in splenic TfR may not be specific to TfRMAbs but rather an IgG-specific response. This increase in splenic-TfR following chronic TfRMAb dosing is also consistent with a recent report showing increased splenic accumulation of TfRMAb-decorated liposomes and gold nanoparticles [27], and a high volume of distribution of a TfRMAb-based fusion protein in the mouse spleen [32].

At the end of the chronic dosing study, all the mice received a single 3 mg/kg SQ dose of TfRMAb to determine the effect of chronic TfRMAb dosing on plasma PK of TfRMAb (Figure 8). The plasma concentration-time profiles showed profound differences in the plasma exposure of the TfRMAb in the different groups and the plasma exposure was the highest in the saline-treated mice that received TfRMAb. Both the mice chronically treated with IgG1 and those treated with TfRMAb had significantly lower TfRMAb plasma exposure compared with the mice chronically treated with saline, and the plasma AUC of TfRMAb was 66% and 97% lower in the IgG1- and TfRMAb-treated mice, respectively. It should be noted that the single TfRMAb dose was injected seven days after the last four-week injection to allow sufficient time for the TfRMAb to be cleared from the blood and to rule out the interference of circulating TfRMAbs on the plasma concentration measurements. This clearance time of seven days however, may not be enough to clear the high circulating levels of mouse IgG1 that are expected to accumulate following chronic IgG1 dosing. The finding of reduced TfRMAb plasma concentrations following chronic TfRMAb dosing was recently reported for a humanized high-affinity bivalent TfRMAb, not fused to a fusion partner, in non-human primates following chronic IV dosing [7]. The reduced plasma concentrations of TfRMAb in the aforementioned study were attributed to accelerated clearance of the antibody, which was saturated at high antibody doses. The contribution of anti-drug antibodies to the reduced plasma concentration was ruled out [7]. One proposed mechanism for enhanced TfRMAb clearance following chronic dosing is via an increase in peripheral TfR levels, and we observed a significant increase in splenic TfR in mice treated with the TfRMAb and IgG1 in the current study. Therefore, an increase in peripheral clearance of TfRMAb mediated by an increase in peripheral TfR levels may explain the reduced plasma exposure in the chronically treated mice. Another mechanism that may be involved in the accelerated clearance of the TfRMAb following chronic dosing is the saturation of the neonatal Fc receptor (FcRn). It has been suggested that increased doses of intravenous immunoglobulins can saturate the “rescue” FcRn and reduce the plasma half-life of antibodies [33]. However, since previous work shows that the accelerated clearance of the TfRMAb is saturated and not enhanced at high injection doses [7], the theory that increased plasma immunoglobulins saturate the FcRn rescue-receptor and accelerate plasma clearance of the TfRMAb is less likely in the current study. It should be noted that studies with a high-affinity bivalent TfRMAb, but fused to a therapeutic protein (GDNF), showed no changes to the plasma PK of the TfRMAb-fusion protein following chronic dosing [19]. This finding is in contrast to our results and those reported for a humanized version of the TfRMAb not fused to a therapeutic protein [7]. Collectively, these results imply that the plasma PK of the TfRMAb may differ depending on the presence or absence of a fusion partner and the overall impact of chronic TfRMAb dosing on plasma PK may depend on the fusion partner involved.

In conclusion, this study provides data on the plasma PK-, hematologic-, iron-indices, and overall health of mice, of a high-affinity bivalent TfRMAb with a murine constant domain, when given at low therapeutic doses following SQ administration, in mice. The results showed an overall favorable hematologic and iron profile following chronic dosing at low therapeutic doses in mice consistent with published work with TfRMAbs. The acute clinical signs seen with humanized or rat TfRMAbs following IV administration in mice were not observed in the current study following SQ dosing. This study highlights some differences (and similarities) in the safety and PK behavior of TfRMAbs based on affinity, dosing regimen, isotype, and/or the fusion partner involved, aspects that may require consideration while developing TfRMAbs as drug delivery vectors in the future.

## Figures and Tables

**Table 1:** PK parameters for TfRMAb following SQ dosing in mice. Data is shown as the mean ± SEM of *n* = 3 per dose.

Route	Dose (mg/kg)	AUC (0–24) (µg·min/mL)	Cmax (µg/mL)	Tmax (min)	Apparent Clearance (mL/min/kg)	Cmax Ratio	AUC Ratio	Clearance Ratio
SQ	3	1268 ± 221	1.5 ± 0.3	360	2.6 ± 0.5	0.66 ± 0.1 ^#^	0.63 ± 0.1 ^$^	0.88 ± 0.2
	5	2016 ± 523	2.3 ± 0.7	300 ± 60	2.9 ± 0.9	1.0 ± 0.3	1.0 ± 0.3	1.0 ± 0.3

PK parameters were normalized to the 5 mg/kg values to give the Cmax, AUC and clearance ratios. ^#^ *p* = 0.07, ^$^ *p* = 0.08.

**Table 2:** PK parameters of TfRMAb following a single 3 mg/kg SQ injection in mice following four-week chronic dosing of saline, TfRMAb or IgG1. Data is shown as the mean ± SEM of *n* = 4 per group.

Treatment Group	AUC (0–24) (µg·min/mL)	Cmax (µg/mL)	Tmax (min)	Apparent Clearance (mL/min/kg)	Cmax Ratio	AUC Ratio	Clearance Ratio
Saline+ TfRMAb	658 ± 196	0.76 ± 0.2	360	6.2 ± 2	1.0 ± 0.3	1.0 ± 0.3	1.0 ± 0.3
IgG1+ TfRMAb	221 ± 29	0.26 ± 0.04	360	14 ± 2	0.34 ± 0.06 **	0.34 ± 0.04 **	2.3 ± 0.3 *
TfRMAb+ TfRMAb	18 ± 8	0.016 ± 0.007	1170 ± 270	332 ± 137	0.020 ± 0.09 **	0.028 ± 0.01 **	54 ± 22 #

PK parameters were normalized to the saline+TfRMAb group to give the Cmax, AUC and clearance ratios. * *p* < 0.05, ** *p* < 0.01, # *p* = 0.09.
